# Spinal Counter Stimulation in Congestive Fever

**Published:** 1841-12

**Authors:** 


### SPINAL COUNTER STIMULATION IN CONGESTIVE FEVER.

Dr. Jno. B. Baird, of Franklin, Ky., writes us as follow: "In this neighborhood, this fall, the fevers were wont to assume a congestive type, and the bowels to be obstinately torpid—the strongest cathartics, repeated from day to day, producing no alvine evacutions. In such cases, sinapisms over the spine, as recommended by Professor Yandell, Professor Caldwell and others, invariably producced the desired effect, if used in time, and those who were treated without them, as certainly died." D.

